# Impaired brain-heart axis in focal epilepsy: Alterations in information flow and implications for seizure dynamics

**DOI:** 10.1162/netn_a_00367

**Published:** 2024-07-01

**Authors:** Lorenzo Frassineti, Vincenzo Catrambone, Antonio Lanatà, Gaetano Valenza

**Affiliations:** Department of Information Engineering, Università degli Studi di Firenze, Firenze, Italy; Department of Information Engineering and Bioengineering & Robotics Research Center E. Piaggio, University of Pisa, Pisa, Italy

**Keywords:** Brain-heart axis, EEG, Heart rate variability, Central autonomic network, Epilepsy, SDG model

## Abstract

This study delves into functional brain-heart interplay (BHI) dynamics during interictal periods before and after seizure events in focal epilepsy. Our analysis focuses on elucidating the causal interaction between cortical and autonomic nervous system (ANS) oscillations, employing electroencephalography and heart rate variability series. The dataset for this investigation comprises 47 seizure events from 14 independent subjects, obtained from the publicly available Siena Dataset. Our findings reveal an impaired brain-heart axis especially in the heart-to-brain functional direction. This is particularly evident in bottom-up oscillations originating from sympathovagal activity during the transition between preictal and postictal periods. These results indicate a pivotal role of the ANS in epilepsy dynamics. Notably, the brain-to-heart information flow targeting cardiac oscillations in the low-frequency band does not display significant changes. However, there are noteworthy changes in cortical oscillations, primarily originating in central regions, influencing heartbeat oscillations in the high-frequency band. Our study conceptualizes seizures as a state of hyperexcitability and a network disease affecting both cortical and peripheral neural dynamics. Our results pave the way for a deeper understanding of BHI in epilepsy, which holds promise for the development of advanced diagnostic and therapeutic approaches also based on bodily neural activity for individuals living with epilepsy.

## Author Summary

This study focuses on brain-heart interplay (BHI) during pre- and postictal periods surrounding seizures. Employing multichannel EEG and heart rate variability data from subjects with focal epilepsy, our analysis reveals a disrupted brain-heart axis dynamic, particularly in the heart-to-brain direction. Notably, sympathovagal activity alterations during preictal to postictal transitions underscore the autonomic nervous system’s pivotal role in epilepsy dynamics. While brain-to-heart information flow targeting low-frequency band cardiac oscillations remains stable, significant changes occur in cortical oscillations, predominantly in central regions, influencing high-frequeny-band heartbeat oscillations, that is, vagal activity. Viewing seizures as states of hyperexcitability and confirming focal epilepsy as a network disease affecting both central and peripheral neural dynamics, our study enhances understanding of BHI in epilepsy. These findings offer potential for advanced diagnostic and therapeutic approaches grounded in bodily neural activity for individuals with epilepsy.

## INTRODUCTION

Epilepsy is a prevalent neurological disorder affecting a significant number of individuals worldwide, as reported by the World Health Organization. It is estimated that approximately 50 million people suffer from epilepsy (World Health Organization, 2024). Unfortunately, around 30% of patients experience resistance to the standard anti-epileptic drugs commonly used to control and minimize the occurrence of seizures (World Health Organization, 2024). Therefore, it remains crucial to gain a comprehensive understanding of the intricate pathophysiological mechanisms underlying this condition. In pursuit of this goal, extensive research has been conducted to characterize the disease’s neurological dynamics (Breakspear et al., 2006; Jirsa, Stacey, Quilichini, Ivanov, & Bernard, 2014), particularly during or in close proximity to seizure events. Additionally, numerous methods have been developed to facilitate automatic detection or prediction of seizure events, aimed at supporting clinical personnel (Ramgopal et al., 2014).

Numerous studies have been conducted to investigate the underlying brain dynamics in individuals with epilepsy (Cherian & Kanaga, 2022; Jirsa et al., 2014; Lehnertz, Bröhl, & von Wrede, 2023; Lehnertz, Geier, Rings, & Stahn, 2017). Notably, there is a growing consensus in the literature, as highlighted by Lehnertz et al. (2023), that epilepsy should be regarded as a network disease. Jirsa et al. (2014) explain that during epileptic seizures, there are well-documented instances of hyperexcitability or hypersynchrony within brain neural networks. However, it is important to note that these conditions cannot be universally generalized to all epileptic syndromes or seizures (Fisher et al., 2014). Additionally, significant research efforts have been dedicated to understanding the dynamics at the onset of seizures (Geier, Bialonski, Elger, & Lehnertz, 2015), as well as unraveling the neurophysiological mechanisms responsible for terminating seizure events (Lado & Moshé, 2008) and exploring the concept of self-perpetuating seizures (Burman, Raimondo, Jefferys, Sen, & Akerman, 2020).

The concept of epilepsy as a network disease has garnered increasing attention, prompting investigations into the intricate interactions between the central nervous system (CNS) and the autonomic nervous system (ANS) in individuals with epilepsy (Costagliola et al., 2021). This avenue of research is closely linked to the assessment of brain-heart axis functioning and related brain-heart interplay (BHI) (Silvani, Calandra-Buonaura, Dampney, & Cortelli, 2016). Recent studies propose that cardiac abnormalities during seizures may directly contribute to the pathogenesis of sudden unexpected death in epilepsy (Costagliola et al., 2021). Accordingly, the significance of heart rate variability (HRV) analysis, reflecting ANS activity, in detecting substantial alterations in individuals with epilepsy is well established (Leal et al., 2021; Verrier, Pang, Nearing, & Schachter, 2020). Particularly in patients with temporal lobe epilepsy (TLE), HRV demonstrates noteworthy changes (Myers, Sivathamboo, & Perucca, 2018). Furthermore, prior research by Jaychandran et al. (2016) reported increased heart rate during the preictal phase leading up to seizure events in a majority of patients. Exploring interictal cardiorespiratory variability in both TLE subjects and children with absence epilepsy, Varon et al. (2015) revealed potential effects of absence epilepsy on the cardiac and respiratory control mechanisms of the ANS. Schiecke et al. (2015) proposed a matching-pursuit-based bispectrum analysis for quantifying quadratic phase coupling in HRV signals from children with TLE. Their findings indicated significant increases in the HRV bispectrum frequencies, with differences between preictal and postictal periods being more pronounced than during the seizure events.

A limited number of studies have been conducted on BHI assessment in individuals with epilepsy. For instance, Schiecke et al. (2016) employed the convergent-cross-mapping approach (Sugihara et al., 2012) to investigate BHIs in 18 children with TLE. Their study revealed significant EEG-HRV interactions, with notable emphasis on the delta (*δ*) and alpha (*α*) frequency bands. In a separate investigation, Frassineti et al. (2022) explored BHIs in newborns with seizures, observing relatively weaker interactions compared with seizure-free patients. These findings suggest the potential utility of HRV and BHI analysis as tools for neonatal seizure detection and characterization (Statello, Carnevali, Sgoifo, Miragoli, & Pisani, 2021). Additionally, Seleznov et al. (2020) employed a multiscale cross-correlation approach to examine the relationship between EEG and HRV signals in focal epilepsy. Notably, they observed significant differences in the delta (*δ*) frequency band (0.5–4 Hz) before and after seizures, indicating the involvement of nonlinear mechanisms in the CNS-ANS interactions. Furthermore, Kassinopoulos, Harper, Guye, Lemieux, and Diehl (2021) investigated the relationship between HRV and functional magnetic resonance imaging in 28 patients with drug-resistant epilepsy and 16 healthy subjects. Their study revealed a decreased inter-beat (RR) interval series and related power in the high-frequency (HF) band in individuals with epilepsy compared with healthy controls. These studies collectively contribute to the understanding of BHI in epilepsy, highlighting the functional relationship between EEG and HRV signals in various seizure contexts. However, more research is warranted to gain deeper insights into the mechanisms underlying these interactions and their potential implications for epilepsy diagnosis and management (Bahari, Ssentongo, Schiff, & Gluckman, 2018; Fujiwara et al., 2016; Kassinopoulos et al., 2021; Pinto et al., 2021). Indeed, despite ongoing research efforts, the intricate mechanisms underlying the CNS-ANS interactions during or near epileptic seizures remain elusive and challenging to monitor or quantify. The quantification of functional BHI has gained increasing interest in recent years. Methodologically, this quantification encounters numerous technical challenges because of its multimodal and multivariate nature. Adding to these challenges are issues related to directionality, wherein brain-to-heart and heart-to-brain interactions may not align, and the demand for use of physiological plausible models, given that conventional signal processing tools may not be well-suited for analyzing such physiological phenomena. Despite these challenges, various techniques have been implemented or specifically developed for BHI estimation. Quantifiers within the framework of information theory have been devised to estimate both linear (Faes et al., 2016) and nonlinear (Faes, Marinazzo, Jurysta, & Nollo, 2015) interactions. Additionally, techniques such as a transfer entropy formulation based on a point process model have been designed to explore how heartbeat dynamics are instantaneously influenced by cortical activity (Catrambone, Talebi, Barbieri, & Valenza, 2021). Conversely, methods like heartbeat-evoked potentials have investigated the overall scalp activity response to the heartbeat, serving as an interoceptive measure (Al et al., 2020). Moreover, specific research has delved into BHI representation in the multifractal domain (Catrambone, Barbieri, Wendt, Abry, & Valenza, 2021), as well as the complexity of cerebrovascular joint dynamics (Catrambone & Valenza, 2023a; Porta, Cairo, De Maria, & Bari, 2020). Recent studies have also focused on detecting microstates (i.e., quasi-stable spatiotemporal states) of brain-heart axis dynamics (Catrambone & Valenza, 2023b, 2023c). While the aforementioned literature highlights the viability and significance of quantifying functional BHI, it has not undergone thorough investigation in epilepsy research. Accordingly, this study suggests quantifying BHIs in epileptic subjects by employing a synthetic data generation (SDG) model designed for a directed BHI assessment (Catrambone, Greco, Vanello, Scilingo, & Valenza, 2019). SDG is a physiologically plausible multivariate model, specifically designed for an ad hoc, fully parametric directional estimation of BHI. We focus specifically on interictal periods preceding and following seizure events and utilize a publicly available dataset that includes multichannel EEG and ECG signals (Detti, n.d.; Detti, Vatti, & Zabalo Manrique de Lara, 2020). Our primary objective is to characterize the BHI temporal dynamics of interictal periods both preceding and following ictal events. We utilize a Wigner-Ville-based method to extract cortical and heartbeat dynamics information in the frequency domain. Furthermore, considering recent findings in the literature, we delve into the potential pathophysiological mechanisms underlying BHI interactions in epilepsy, along with relevant neurophysiological discoveries. This discussion aims to provide insights into the direct or indirect involvement of the ANS in epileptic subjects.

## MATERIAL AND METHODS

### Siena Scalp EEG Database

In this study, the publicly available Siena Scalp EEG Database (Detti, n.d.; Detti et al., 2020), hereinafter referred to as the Siena Dataset, was utilized. The dataset comprised recordings from 14 subjects (9 males and 5 females) within an age range of 20 to 71 years. These recordings were collected at the Unit of Neurology and Neurophysiology of the University of Siena, Italy. Simultaneous EEG and ECG signals were acquired for each subject, with a total recording length of 128 hr and a sampling frequency of 512 Hz. All subjects had a confirmed diagnosis of epilepsy, and at least one seizure event was detected and expertly labeled by clinicians for each recording (for more detailed information, please refer to Detti, n.d.). The dataset contained a total of 47 seizure events. Among the subjects, the majority exhibited focal left temporal seizures (9 subjects), while 4 subjects had focal right temporal seizures, and 1 subject experienced focal to bilateral tonic-clonic seizures. All subjects had at least one ECG derivation. The EEG derivations employed in this study included the following channels: Fp1, F3, C3, P3, O1, F7, T3, T5, Fc1, Fc5, Cp1, Cp5, F9, Fz, Cz, Pz, Fp2, F4, C4, P4, O2, F8, T4, T6, Fc2, Fc6, Cp2, Cp6, and F10. However, for subject PN10, the following 10 derivations were not recorded: Fc1, Fc5, Cp1, Cp5, F9, Fc2, Fc6, Cp2, Cp6, and F10. For all subjects, 29 EEG channels were considered for further analysis, except for subject PN10, where 19 EEG channels were used. All methods presented in this work were implemented using MATLAB software, specifically version 2021b.

### Signal Preprocessing

To ensure signal quality, each EEG derivation was filtered using a band-pass FIR filter with cutoff frequencies of 1–32 Hz. For the extraction of R-peaks and the corresponding HRV signal, the ECG recordings were analyzed using Kubios software (version 2.2; Tarvainen, Niskanen, Lipponen, Ranta-Aho, & Karjalainen, 2014). Prior to analysis, a medium artifact correction was applied, and visual inspection was performed to identify and remove any remaining artifacts. The resulting RR interval signals were interpolated using the cubic method and resampled to a frequency of 4 Hz.

For each seizure event, a 10-min window preceding the labeled clinical onset and a 10-min window following the labeled clinical offset were extracted for both the EEG and HRV signals. These two interictal periods are hereinafter referred to as the preictal and postictal periods, respectively. It is important to note that these designations were chosen purely for ease of identification with respect to the seizure event and do not imply the presence of precursors or the duration of the postictal state. They were indeed considered to be interictal windows occurring before and after each seizure event and were given the same duration. The choice of a 10-min window was a compromise between reliable estimation of BHIs (Shaffer & Ginsberg, 2017; Valenza et al., 2016) and the amount of available data for each subject. Consequently, this study included data from 13 subjects, as subject PN12 was excluded because of the limited amount of pre- and postictal data available. After these preprocessing steps, a total of 38 seizure events from the 13 included subjects were evaluated.

### Directional Brain-Heart Interplay Assessment

The synthetic data generation (SDG) model (Catrambone et al., 2019) was employed in this study to examine the bidirectional modulations between EEG oscillations within a specific frequency band and heartbeat dynamics spectra integrated over low- or high-frequency bands. EEG frequency bands were as follows: *δ* (1–4 Hz), *θ* (4–8 Hz), *α* (8–12 Hz), and *β* (12–30 Hz). HRV frequency bands were as follows: low frequency (LF, 0.04–0.15 Hz), high frequency (HF, 0.15–0.4 Hz).

The SDG model quantifies the functional interplay from the brain to the heart by generating synthetic heartbeat intervals based on an integral pulse frequency modulation (IPFM) model, parameterized using Poincaré plot features. The synthetic heartbeats are generated based on a reference heart rate and a modulation function *m*(*t*).

$$m\left(t\right)=C_{LF}\left(t\right)\sin\left(\omega_{LF}t\right)+C_{HF}\left(t\right)\sin\left(\omega_{HF}t\right).$$ (1)

Here, *ω*_*LF*_ and *ω*_*HF*_ are the central frequencies associated with each HRV frequency band, and *C*_*i*_ (for *i* = *LF*, *HF*) represent the activity of the associated band. *C*_*i*_(*t*) can be formalized as follows:

$$C_{i}\left(t\right)=C_{i}\left(0\right)+{SDG}_{\text{brain}\to i}\left(t^{-}\right)\times P_{\text{brain}}\left(t^{-}\right),$$ (2)

where *P*_brain_(*t*^−^) quantifies the activity of one of the bands considered in the EEG analysis at time instant *t*^−^ preceding *t*. The coefficients *SDG*_brain→*i*_ (i.e., *SDG*_brain→*LF*_ and *SDG*_brain→*HF*_) represent the brain-to-heart interplay, while the LF and HF powers correspond to sympathovagal and parasympathetic dynamics, respectively. To quantify the functional interplay from the heart to the brain, a model based on an adaptive Markov process is employed (Catrambone et al., 2019). This model estimates ascending modulations from the heart to the brain using least squares in an autoregressive process. The Markovian neural activity generation, specific to an EEG channel, frequency band, and time window, utilizes the previous neural activity and the current heartbeat dynamics as inputs.

$$EEG\left(t\right)=\sum_{j=1}^{K}a_{j}\left(t\right)\sin\left(\omega_{j}t+\phi_{j}\right).$$ (3)

Here, *ω*_*j*_ is the main oscillation associated with each of the *K* = 4 EEG frequency bands (*j* ∈ {*δ*, *θ*, *α*, *β*}). The term *a*_*j*_(*t*) represents the *j*th band activity and is modeled as follows:

$$a_{j}\left(t\right)=\eta_{j}a_{j}\left(t^{-}\right)+{SDG}_{i\to j}\left(t^{-}\right)\times P_{i}\left(t^{-}\right).$$ (4)

Besides the autoregressive term *η*_*j*_*a*_*j*_(*t*^−^), the heart-to-brain coupling is made explicit (i.e., *SDG*_*i*→*j*_, with *i* ∈ *LF*, *HF*), directly modulating the interaction that the activity of the *i*th HRV band (quantified through *P*_*i*_(*t*^−^)) exercises on the *j*th EEG band.

For clarity, hereinafter we utilize the acronyms *BtH* and *HtB* to indicate the two opposite directions of interaction, representing brain-to-heart and heart-to-brain, respectively. For example, *BtH θ* → *HF* refers to the statistical test performed on theta (*θ*) waves and HRV high frequency (HF), indicating the brain-to-heart interplay. The source code implementing the SDG framework for functional BHI is available at https://github.com/CatramboneVincenzo/Brain-Heart-Interaction-Indices.

### Statistical Analysis and Topographic Representation of BHI Values

In this study, we aimed to assess potential differences in functional BHI between preictal and postictal periods. A normality test was performed for each BHI sample using the Shapiro-Wilk test. Since the normality assumption was violated (level of significance 0.05) for the majority of samples associated with preictal and postictal phases, further statistical comparisons are based on nonparametric analyses. To perform this analysis, the nonparametric statistical Wilcoxon signed-rank test was used, with a significance level *α* = 0.01. The test was applied to all considered EEG derivations. To mitigate false discovery rates, a spatial cluster permutation correction was implemented, setting the cluster size to 2 (Friston, Worsley, Frackowiak, Mazziotta, & Evans, 1994). To provide a whole-scalp overview, statistical results were reported as topographic representations.

To gain further insights about the BHI dynamics during the preictal and postictal periods, each 10-min window was divided into 10 subsequent 1-min windows, thus providing a time representation of BHI trends. For each 1-min window, BHI values were averaged across time, taking the median value. Additionally, the ten 1-min window BHI averages related to each seizure were z-scored, thus making the different seizures comparable among them. Finally the obtained z-scored BHI estimates were averaged across the 38 seizures to evaluate the BHI dynamics from the preictal to postictal phase.

## RESULTS

**Figure 1.**
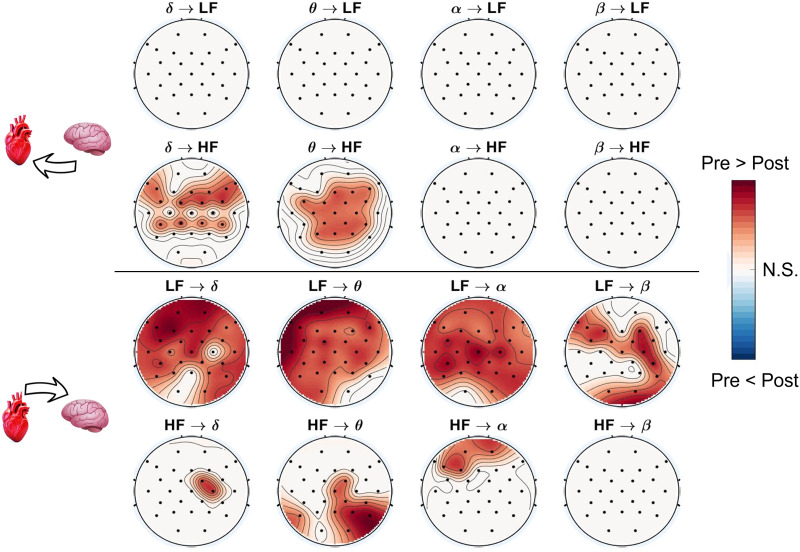
Topographic maps representing statistical results of the BHI analysis comparing preictal and postictal periods. The first two rows are related to *BtH* interactions, for HRV-LF (first row) and HRV-HF (second row) bands and for the brain waves examined (i.e., *δ*, *θ*, *α*, and *β*, represented on the four columns). The last two rows stand for *HtB* interactions. White areas are not significant (N.S.), whereas red regions indicate significantly higher preictal values compared with postictal values, and blue areas indicate the opposite. Darker colors are related to lower *p* values (4.07*e*; five minimum detected).

Figure 1 depicts the statistically significant differences in BHI values between the preictal and postictal periods. The first two rows report on the *BtH* interactions, considering both the LF (first row) and HF (second row) components for all four brain waves examined (i.e., *δ*, *θ*, *α*, and *β*, represented on the four columns). The last two rows illustrate the statistical results related to the *HtB* interactions. Each topographic map displayed the statistically significant regions enhanced by the Wilcoxon test. White areas are not significant, whereas red regions indicate significantly higher preictal values compared with postictal values, and blue areas indicate the opposite. Notably, darker areas are related to lower *p* values (4.07*e*; five minimum detected).

Specifically, looking at the HtB interplay (lower two rows in Figure 1), the HRV-LF component exhibits several significant brain regions, particularly on *δ*, *θ*, and *α* brain waves. It is noteworthy that they can be considered to be generalized, since most of the EEG derivations are involved. Moreover, *LF* → *β* differences between preictal and postictal periods are located mainly in the right hemisphere (central and occipital regions), and in the left frontal area. To sum up, the HRV-LF component in HtB interplay shows the most diffuse significant differences, in terms of both brain waves and derivations, especially for the case *LF* → *δ* and *LF* → *θ* (where the minimum *p* values were detected).

**Figure 2.**
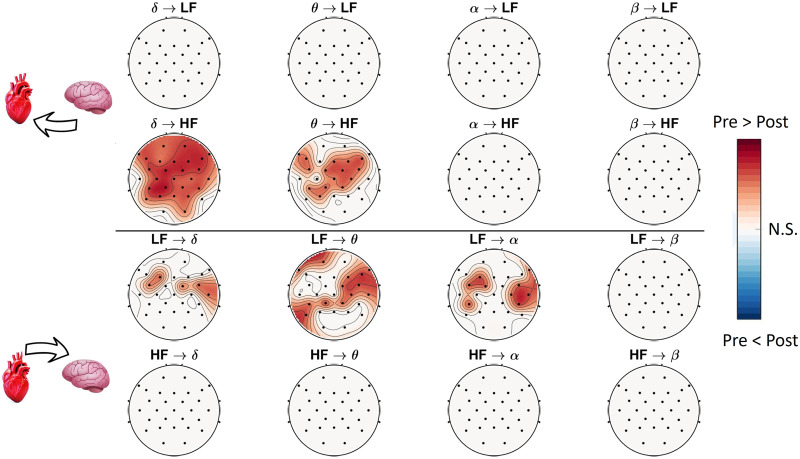
Topographic maps illustrating the statistical results of the BHI analysis, comparing preictal and postictal periods using 5-min windows.

On the other hand, considering the HRV-HF component of the HtB interplay, significant differences between preictal and postictal periods are found to be less spread than the LF cases. Mainly they can be found in the *HF* → *θ*, located on the right posterior hemisphere (see Figure 1). Looking at the *HF* → *α* case, significant differences can be found in the frontal regions. Conversely, in the opposite direction, the BtH cases show less significant differences than the HtB ones. Specifically, such differences are exclusively related to the *δ* → *HF* and *θ* → *HF* cases, both concentrated in the central brain regions. Of note, no significant difference is found between preictal and postictal periods for all the brain waves and EEG derivations considered in the brain-to-LF interplay. In a further analysis, windows shorter than 10 min were assessed (i.e., 7, 5, 3, 2, and 1 min). The main functional direction of BHI remained consistent across varying time windows, supporting the robustness of our findings based on the SDG method. On the one hand, we found that comparable alterations in BHI can be observed through 5-min windows or more, as reported in Figure 2. On the other hand, BHI differences were less pronounced for windows shorter than 5 min. Detailed results on the statistical analysis for all the time windows are reported in the Supporting Information (see Figure S1).

**Figure 3.**
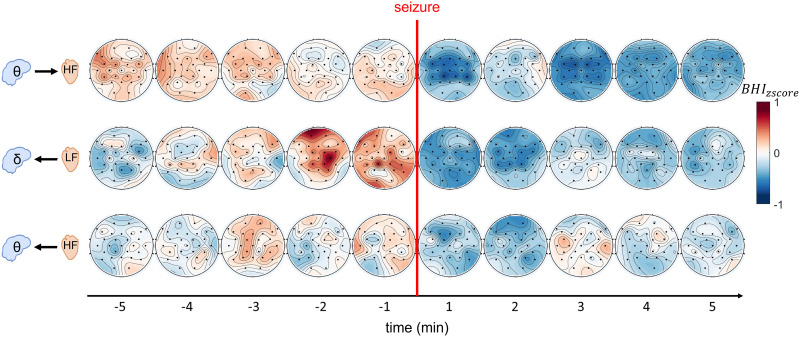
BHI spatiotemporal dynamics through topographic representation. The first row reports the BtH *θ* → *HF*, the second is for the HtB *LF* → *δ*, and the last row represents the HtB *HF* → *θ* dynamics.

To provide insight into the spatiotemporal dependencies of BHI, Figure 3 illustrates selected examples of BHI dynamics across the scalp (obtained as explained in the section called Statistical Analysis and Topographic Representation of BHI Values). The complete set of temporal dynamics for all BHI comparisons is available in the Supporting Information (Figure S2). Rows illustrate the corresponding BHI dynamics across the scalp, as detailed in Figure 1 and explained in the section Statistical Analysis and Topographic Representation of BHI Values. Panel A depicts the BtH dynamics, while panel B showcases the HtB dynamics.

Specifically, the last five 1-min windows preceding the seizure events and the first five 1-min windows following the seizure events are shown. The first row reports the BHI trends for the *θ* → *HF* bands. It is notable that the preictal values are consistently higher than the postictal ones. This trend is enhanced on the central region of the scalp, which indeed was reported to be significant in Figure 1. Notably, the higher values are detected in the first 3 min (i.e., from 5 to 3 min before seizure), then a decrease starts and the first postictal minute highlights lower values. After a rebound 2 min after the seizure, from the third postictal minute on, *θ* → *HF* BHI estimates remain low. In the middle trend in Figure 3 (BHI *LF* → *δ*), it is evident that the BHI values consistently decrease in the postictal windows leading up to the seizure events (indicated by the red line), while the postictal windows exhibit lower BHI values compared with the preictal ones. Specifically, the highest values can be found in the preictal periods in the last two time windows (from 2 min to the seizure), and no initial rebound during the first windows of the postictal periods is detected. Furthermore, in the lower row of Figure 3 the trend of BHI values for the *HF* → *θ* interaction are depicted. In this case, the most evident difference between preictal and postictal phases can be found close to the seizure events: from last 3 preictal minutes to the second postictal window. This confirms the significant differences reported in Figure 1 (last row, second topographic map), where the highest values can be found in the central-posterior area of the right hemisphere. In recent literature, there has been a debate on the concept of lateralization of autonomic control in the brain for individuals with epilepsy (Dono et al., 2020; You et al., 2023). Considering that BHI analysis has previously demonstrated lateralization in the brain (Greco et al., 2019), even if not explicitly in the context of epilepsy, our results indicate that differences in BHI were primarily located on the right hemisphere. These findings suggest that BHI analysis may unveil a general lateralization shift toward the right hemisphere associated with ictal events. Interestingly, during the postictal phase, BHI values tend to be at the same level of the preictal ones starting 3 min after the seizure events, especially on the left and right temporal lobe.

Finally, within the Supporting Information, we have included two additional analyses to support the associations between impaired brain-heart axis leading to BHI functioning disruption and focal epilepsy. Both evaluations utilized the same statistical analysis presented in Figure 1. The first analysis is detailed in Supporting Information Figure S3, focusing on BHI analysis between two interictal periods occurring at a distance from the first ictal event for each recording. In this instance, 5-min windows were employed, selecting periods 1 hr before the first ictal event, considering a total of 28 events owing to the available interictal data in the Siena Dataset. The 5-min windows were separated by 1 min to simulate a surrogate ictal event. Notably, no statistical differences were identified in this case. In contrast, the second analysis pertained to BHI analysis between distant interictal periods, preictal, and postictal periods, with results reported in Figure S4. For this analysis, interictal 10-min windows 1 hr before the first ictal events were utilized. The findings indicated limited BHI differences between preictal and interictal periods (Figure S4a), while several differences emerged between interictal and postictal periods (Figure S4b). These outcomes reinforce the possibility that these differences may be specifically linked to BHI alterations arising from ictal events. It is essential to note that, for clarity, these evaluations cannot definitively confirm the relationship between BHI disruption and impaired brain-heart axis functioning, necessitating further studies. For instance, because of the absence of postictal data in the Siena Dataset, we were unable to evaluate interictal periods occurring 1 hr after the seizure events. Nevertheless, these assessments may be considered to be a preliminary indication of the connections between BHI disruption and brain-heart axis functioning.

## DISCUSSION

This study delves into the spatiotemporal variations in BHI during interictal periods, both before and after seizure events. To comprehensively assess BHI, we employed the physiologically plausible SDG model (Catrambone et al., 2019), effectively quantifying the functional CNS-ANS interplay through concurrent EEG and HRV time-frequency analysis. A statistical analysis scrutinized interictal dynamics related to consistent 38 seizure events from 13 subjects diagnosed with epilepsy, exploring various levels of spatial and time resolution, including the representation of generalized differences in brain areas through topographic maps (Figure 1).

Our experimental findings uncovered noteworthy differences in specific brain waves associated with heartbeat oscillations. Particularly, the most compelling results emerged in the heart-to-brain cases, where a widespread and highly significant interaction between the CNS and ANS was observed, predominantly concentrated in the HRV-LF frequency band (see Figure 1). These intriguing findings strongly suggest that the transfer of information from sympathovagal dynamics to cortical dynamics undergoes substantial alterations following the occurrence of a seizure event. Evidently, postictal interactions were notably lower than preictal ones, indicating a disruption in the BHI pattern following seizures, which may be indicative of impaired brain-heart axis functioning associated with epilepsy. Conversely, the brain-to-heart dynamics did not exhibit any significant differences in LF frequencies for all brain waves (Figure 1; all topographic maps show nonsignificant values). This implies that the transfer of information from the CNS to the LF component of ANS system activity remains unaltered by the seizure event, suggesting a distinct pattern of interaction in this context.

Recent findings have indicated a potential connection between a disruption in BHI and seizure events, occurring either after or in proximity to the seizures (Costagliola et al., 2021; Schiecke et al., 2016). These studies propose a conceptualization of the CNS epileptic network and the ANS as an interconnected system. Furthermore, the compromised brain-heart functioning may be attributed to acquired or inherited dysfunctions in the ANS and the heart resulting from seizures or epilepsy (Giussani et al., 2023; Li, O’Brien, Todaro, & Powell, 2019; Ravindran, Powell, Todaro, & O’Brien, 2016). Consequently, observed BHI differences could be indicative of these underlying alterations. In contrast, we observed significant differences in the brain-to-heart direction, particularly concerning heartbeat oscillations in the HF band, which is well-established to primarily reflect vagal activity and originates predominantly in central regions, specifically *δ* → *HF* and *θ* → *HF* brain wave interactions (Figure 1, upper topographic maps). Additionally, within the heart-to-brain (HtB) cases, we identified noteworthy differences in the *θ* brain waves (parietal and occipital regions) and *α* brain waves (frontal regions) in relation to HRV-HF frequencies. It is widely recognized that HF frequencies play a crucial role in regulating various physiological processes, such as respiration and vagal tone modulation (Rajendra Acharya, Paul Joseph, Kannathal, Lim, & Suri, 2006; Shaffer & Ginsberg, 2017). These results, in line with previous findings in Costagliola et al. (2021), further support the notion that seizures can disrupt this regulatory behavior shortly after the ictal event. Within the framework of the epileptic brain network, the identified BHI differences concerning brain waves may indeed be associated with specific functional roles attributed to these waves. Alterations in delta and theta activity have been previously linked as markers of the epileptic network (Sip, Scholly, Guye, Bartolomei, & Jirsa, 2021; Tao et al., 2011), while changes in alpha rhythm have been associated with seizure control in epilepsy (Abela et al., 2019). Similarly, studies on functional brain connectivity have demonstrated that epileptic networks may extend to diverse areas, including parietal or central regions (de Campos, Coan, Lin Yasuda, Casseb, & Cendes, 2016; Maccotta et al., 2013). Consequently, alterations in BHI dynamics may manifest in areas not directly implicated in the epileptic focus. Taken together, our study provides critical insights into the complex dynamics of BHI in epilepsy, highlighting the significant impact of seizure events on the brain-heart axis functioning. These findings support the notion of epilepsy as a condition associated with impaired brain-heart axis communication, warranting further research to elucidate the underlying mechanisms and potential implications for diagnostic and therapeutic interventions. Because of the limited amount of time available for some subjects in the postictal phase, it is not possible in this study to confirm whether the postictal values return to the level of the preictal ones or if they reach a different plateau specific to another interictal phase (Fisher & Engel, 2010). Consequently, it cannot be determined whether the observed alterations in BtH interactions at HRV-HF frequencies are indicative of specific precursors during the preictal period that could serve as signs of an impending seizure event. The reasons for the observed differential and more pronounced impairment in the heart-to-brain direction, compared with the brain-to-heart one, could be manifold. This impairment might stem from the seizure’s impact on the central autonomic network, potentially resulting in prolonged alterations in ANS dynamics during the postictal phase. The underlying pathophysiological or etiological factors could be diverse, including channelopathies (Li et al., 2019), fluctuations in catecholamine levels (Nass et al., 2019), among others. However, a detailed investigation into these factors extends beyond the objectives of the presented work. In essence, the occurrence of a seizure event may result in a “reset” of the information flow between the CNS and the ANS. This concept aligns with the notion that epileptic seizures represent a state of hyperexcitability within the nervous system (Jirsa et al., 2014) and are characterized as network diseases (Lehnertz et al., 2023). As a result, these alterations have the potential to not only propagate within the CNS but also have an impact on the ANS (Costagliola et al., 2021; Statello et al., 2021).

Furthermore, as depicted in Figure 1, it is worth noting that the observed BHI differences were widespread, despite the focal nature of the seizures being analyzed. For instance, significant differences were found in almost all the EEG derivations considered for the HtB *LF* → *θ* interaction. A possible speculative explanation regarding the generalization of observed BHI changes could be related to the conceptualization of epilepsy as a network disease (Lehnertz et al., 2023), analogous to BHI being recognized as a physiological network phenomenon (Catrambone, Barbieri, et al., 2021). Consequently, both cortical and subcortical brain regions may be directly or indirectly involved in the epileptic brain network, even in cases of focal seizures. The central-autonomic network, which modulates the interplay between ANS and CNS, is also hypothesized to be implicated in dynamics during or close to ictal events. For these reasons, even focal seizures may produce generalized alterations in BHI dynamics close to the ictal events. Indeed, the topographic trends illustrated in Figure 3 reveal that BHI values exhibited consistent patterns during the preictal phase compared with the postictal phase, when examined in meaningful EEG and HRV frequency ranges. In Figure 3, a constant increase in interactions can be observed for the HtB *LF* → *δ* case, while the case *θ* → *HF* demonstrates that the preictal interaction values are generally higher than the postictal values, across all the 1-min windows considered. These findings partially align with previous studies (Costagliola et al., 2021; Schiecke et al., 2016), which have underscored the functional link between the brain and the heart during seizures, along with the specific alterations occurring in both physiological systems during preictal and postictal periods. In their study, Schiecke et al. (2016) utilized the convergent cross mapping approach and identified directed interactions between HRV and delta-related EEG activity in children with TLE. They also reported interactions between HRV and alpha activity. Another study by Pernice et al. (2022), employing Granger causality and a partial information decomposition approach on 18 children with TLE, found interactions on delta and alpha activity. However, it is important to note that both of these studies focused on younger cohorts than the Siena Dataset, and they limited their BHI analysis to only two EEG channels. Consequently, any direct comparison with these studies should be approached with caution. It is important to highlight that in the present work, the theta EEG band was found to be associated with various cardiovascular dynamics quantifiers, including both HF and LF powers, which is consistent with findings in previous studies (You et al., 2023). Nevertheless, it is essential to emphasize that literature on quantitative BHI in focal epilepsy is still limited, and further studies are highly warranted. These results also indicate the potential for analyzing BHI trends over longer periods to investigate whether such patterns persist throughout the postictal phase or whether they tend to return to preictal values. For clarity, the BHI alterations identified in the postictal phase may represent two distinct phenomena. They could signify a transient state of autonomic dysregulation following seizures (Giussani et al., 2023; Li et al., 2019; Senapati et al., 2023), or they might indicate the onset of a more enduring alteration in BHI. Because of the absence of postictal data in the Siena Dataset, resolving this ambiguity is not feasible. Future studies should explore the evolution of BHI dynamics over extended periods to gain insights into the potential duration and recovery timeline for each subject. It is important to note that not all brain waves exhibited significant differences. As shown in Figure 1, this is particularly evident for the *β* waves, which only showed a few significant differences in the HtB LF case. This suggests that the processes involved in the generation of beta waves (Sherman et al., 2016) may not be significantly influenced by BHI during these preictal or postictal phases. Previous works already reported as low-frequency EEG bands (mainly *δ* and *θ*) were associated with cardiovascular control and heart beat dynamics (Catrambone, Messerotti Benvenuti, Gentili, & Valenza, 2021), as well as how different mechanisms in the heart or ANS may influence specific frequencies in the EEG (Jung, Jang, & Lee, 2019). Moreover, this study primarily focused on analyzing focal temporal epileptic seizures, which have previously shown alterations in HRV signals and BHIs in other cohorts (Leal et al., 2021; Schiecke et al., 2016). Therefore, further investigations are needed to determine whether these interactions are specific to this type of epileptic seizure or whether they can be considered a general pattern of BHI in individuals with epilepsy. Additionally, the cohort studied predominantly consisted of adults, and additional research is required to ascertain whether similar differences in BHI can be observed in children or newborns (Frassineti et al., 2022).

We acknowledge certain limitations in this work. First, the study involves a limited number of seizure events and subjects, focusing primarily on a specific type of epileptic seizure (i.e., TLE). Consequently, the findings regarding BHI differences cannot be directly exploited to different types of epilepsy, such as generalized seizures. Moreover, further investigations are necessary, considering varied age ranges (e.g., pediatric or newborn subjects), to assess the potential impact of age on BHI in epileptic individuals. To validate the results, it is necessary to replicate the methods on other datasets (Obeid & Picone, 2016; Stevenson, Tapani, Lauronen, & Vanhatalo, 2019). Future studies should evaluate the effect of medications on BHI dynamics close to the ictal event in epileptic subjects. Additionally, as explained in the Signal Preprocessing section, the chosen time length for the preictal and postictal windows was determined to strike a balance between maintaining an adequate number of seizure events and obtaining reliable estimations of the HRV spectrum (Catrambone et al., 2019; Shaffer & Ginsberg, 2017). Future research should explore different window lengths to investigate dynamics further away from the seizure event. Another interesting analysis could involve the investigation of BHI during the seizure event itself. In this study, because of movement artifacts and unreliable ECG or EEG recordings during the ictal phase, it was not possible to conduct such analysis for several subjects. However, based on the promising results obtained, it can be inferred that a difference between the preictal and postictal phases occurs, as already suggested in the literature (Costagliola et al., 2021; Verrier et al., 2020). Furthermore, the proposed methods provide a quantitative estimation of the interactions between the CNS and ANS, as well as how these connections vary over time, showing an interesting decrease after the seizure event. The identification of impaired brain-heart axis functioning underscores the intricate and bidirectional interactions between the CNS and the ANS in focal epilepsy. These findings carry significant clinical implications, as disrupted BHI dynamics may contribute to the dysregulation of physiological processes and autonomic function observed in epilepsy. Understanding the mechanisms underlying these disruptions could open avenues for developing targeted interventions aimed at restoring normal brain-heart axis functioning and enhancing the overall management of focal epilepsy. Furthermore, BHI dynamics may play a role in various contexts, including seizure prediction (Lehnertz et al., 2023), sudden unexpected death in epileptic patients (Costagliola et al., 2021; Schiecke et al., 2016), autonomic dysfunction during the postictal phase (Senapati et al., 2023), and the characterization of cardiovascular damage due to epilepsy (Asatryan, 2021). Our study contributes to the growing body of evidence emphasizing the significance of investigating BHI dynamics in epilepsy. A comprehensive analysis of spatiotemporal variations in BHI provides a deeper understanding of the disorder’s pathophysiology and supports the notion of epilepsy as a network disease affecting both cortical and autonomic dynamics.

## CONCLUSION

In conclusion, our study provides valuable insights into the spatiotemporal variations of the functional BHI in focal epilepsy. Through the application of the SDG approach, we investigated the dynamic CNS-ANS interactions during interictal periods, both before and after seizure events. Our findings reveal significant disruptions in the brain-heart axis functioning, particularly evident in the heart-to-brain direction, which are characterized by a widespread and substantial interaction between the CNS and ANS, primarily concentrated in the HRV-LF frequency band. These observed alterations in BHI suggest that the transfer of information from sympathovagal dynamics to cortical dynamics is significantly impacted by seizure events. Notably, postictal interactions were lower than preictal ones. Additionally, our results highlight significant differences in the brain-to-heart direction, particularly concerning heartbeat oscillations in the HF band, which reflects vagal activity and originates predominantly in central brain regions. However, the potential mechanisms behind the observed disruption in BHI remain speculative because of the limited clinical and physiological information available in the Siena Dataset. To bridge this gap, future research should conduct additional analyses in conjunction with BHI approaches. This may involve evaluating blood markers of cardiac stress after seizures (Nass et al., 2019) or incorporating genetic analysis (Li et al., 2019) to gain deeper insights into the underlying factors influencing BHI dynamics. Further research in this area is crucial to unravel the intricate complexities of the brain-heart axis in epilepsy, with the ultimate goal of advancing diagnostic and therapeutic approaches for individuals living with this condition. By addressing the impairments in the brain-heart axis, we can potentially improve the overall quality of life for individuals with focal epilepsy and contribute to better disease management and patient care.

## SUPPORTING INFORMATION

Supporting information for this article is available at https://doi.org/10.1162/netn_a_00367.

## AUTHOR CONTRIBUTIONS

Lorenzo Frassineti: Conceptualization; Data curation; Formal analysis; Investigation; Methodology; Software; Visualization; Writing – original draft. Vincenzo Catrambone: Conceptualization; Formal analysis; Methodology; Software; Validation; Visualization; Writing – review & editing. Antonio Lanatà: Funding acquisition; Investigation; Project administration; Resources; Supervision; Validation; Writing – review & editing. Gaetano Valenza: Conceptualization; Funding acquisition; Project administration; Supervision; Writing – review & editing.

## FUNDING INFORMATION

Lorenzo Frassineti, European Commission (https://dx.doi.org/10.13039/501100000780), Award ID: Horizon 2020 project EXPERIENCE No. 101017727. Lorenzo Frassineti, Italian Ministry of Education and Research, Award ID: FoReLab project. Lorenzo Frassineti, PNRR project THE, Award ID: ECS00000017 - CUP I53C22000780001.

## Supplementary Material



## TECHNICAL TERMS

Brain-heart interplay (BHI): BHI refers to the interactions and related factors that affect the activity between the autonomic and central nervous system.
Heart rate variability (HRV): A measure of the variation or fluctuations in time between consecutive heartbeats.
Temporal lobe epilepsy (TLE): A focal epilepsy that starts in the temporal lobe area of the brain.
Wigner-Ville distribution: A method to represent a signal in the time-frequency domain.
Integral pulse frequency modulation model: A model that generates synthetic inter-beat intervals series with cardiac sympathetic and vagal dynamics as inputs.
Poincaré plot: A geometrical technique usually used to quantify self-similarity or fluctuations in biomedical signals.
Markovian neural activity generation: A method that uses an adaptive Markov process to characterize the variation on neural activity.
BHI lateralization: When the BHI tends to occur primarily over one hemisphere or in a specific brain area.
Vagal tone: The biological process that identifies the activity of the vagus nerve, a component of the autonomic nervous system.
Epileptic brain network: The brain regions involved in the epileptic activities (generation or propagation), across different levels of spatial and temporal scales.
