# Supplementary material for: Impaired brain-heart axis in focal epilepsy: Alterations in information flow and implications for seizure dynamics
Source: Netw Neurosci. 2024 Jul 1;8(2):541–56. doi: 10.1162/netn_a_00367 (PMC11168720; doi:10.1162/netn_a_00367)
Supplement: Supplementary file 1 [file netn-8-2-541-s001.pdf]

Supplementary Materials

Impaired Brain-Heart Axis in Focal Epilepsy: Alterations in Information Flow and Implications for Seizure Dynamics.

Lorenzo Frassineti[1], Vincenzo Catrambone[2], Antonio Lanatà[1], and Gaetano Valenza[2]

[1]Department of Information Engineering, Università degli Studi di Firenze, Via Santa Marta 3, 50139, Firenze, Italy .[2]Department of Information Engineering and Bioengineering & Robotics Research Center E. Piaggio, University of Pisa, Largo Lucio Lazzarino, 1, Pisa, 56126, Italy.

Figure S1 illustrates topographic maps comparing BHI in preictal and postictal periods while varying the time window. The following time windows were specifically evaluated: (a) 7 minutes, (b) 3 minutes, (c) 2 minutes, and (d) 1 minute.

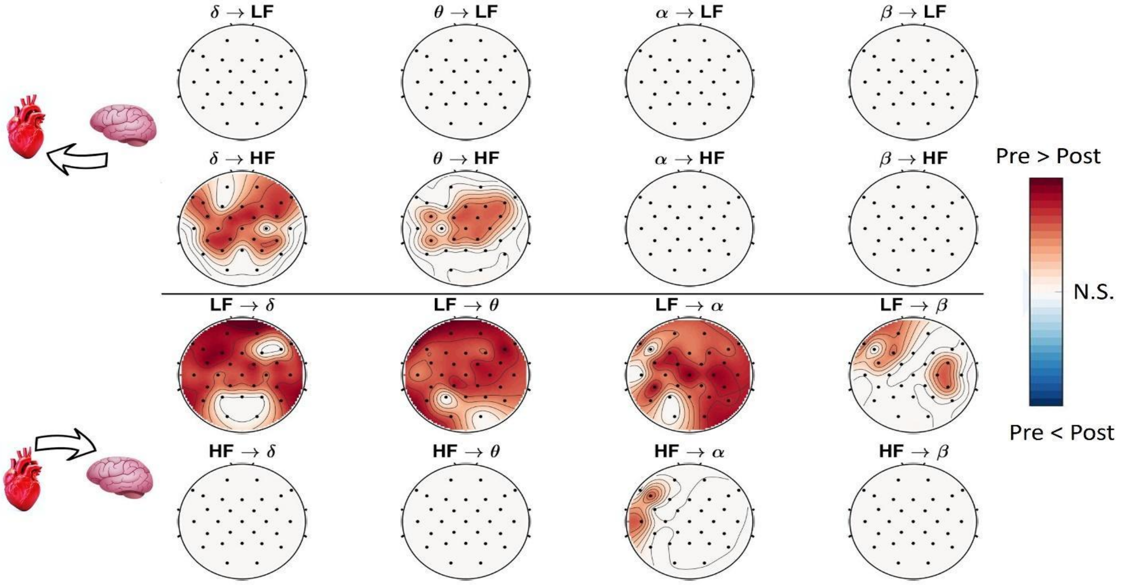

(a). Topographic maps illustrating the statistical results of the BHI analysis, comparing preictal and postictal periods using 7-minute windows.

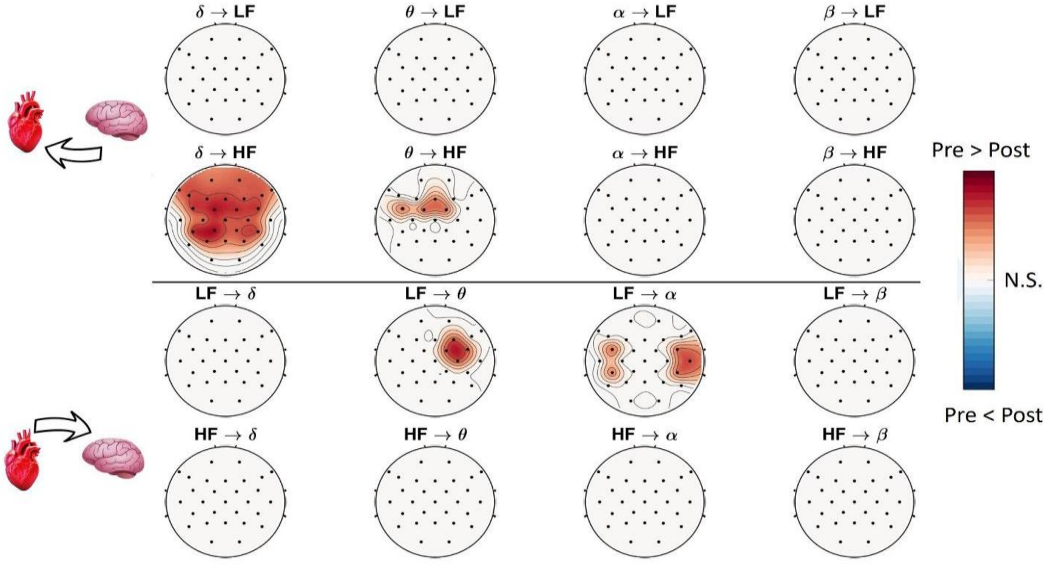

(b). Topographic maps illustrating the statistical results of the BHI analysis, comparing preictal and postictal periods using 3-minute windows.

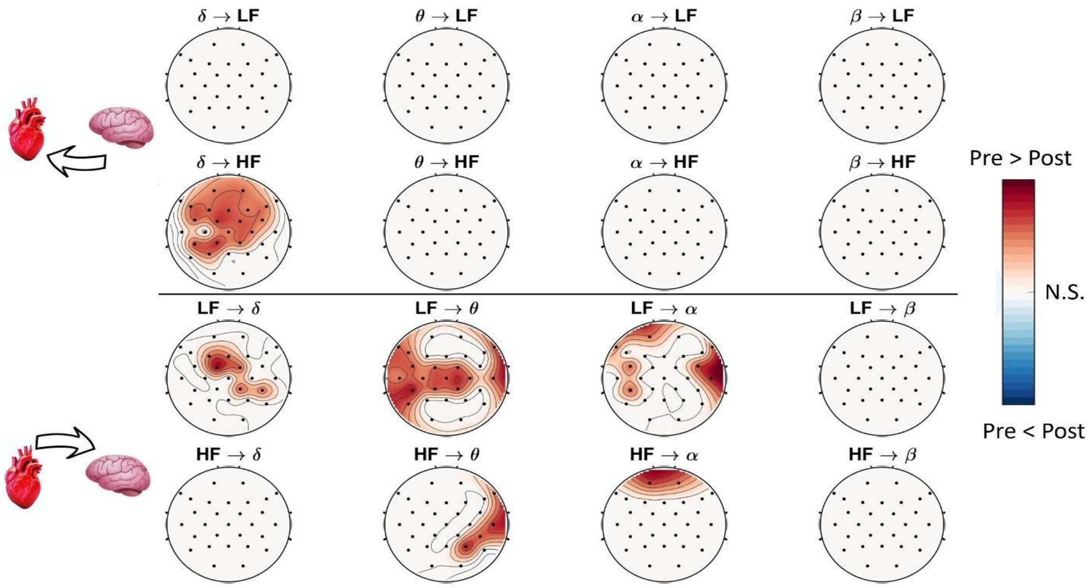

(c). Topographic maps illustrating the statistical results of the BHI analysis, comparing preictal and postictal periods using 2-minute windows.

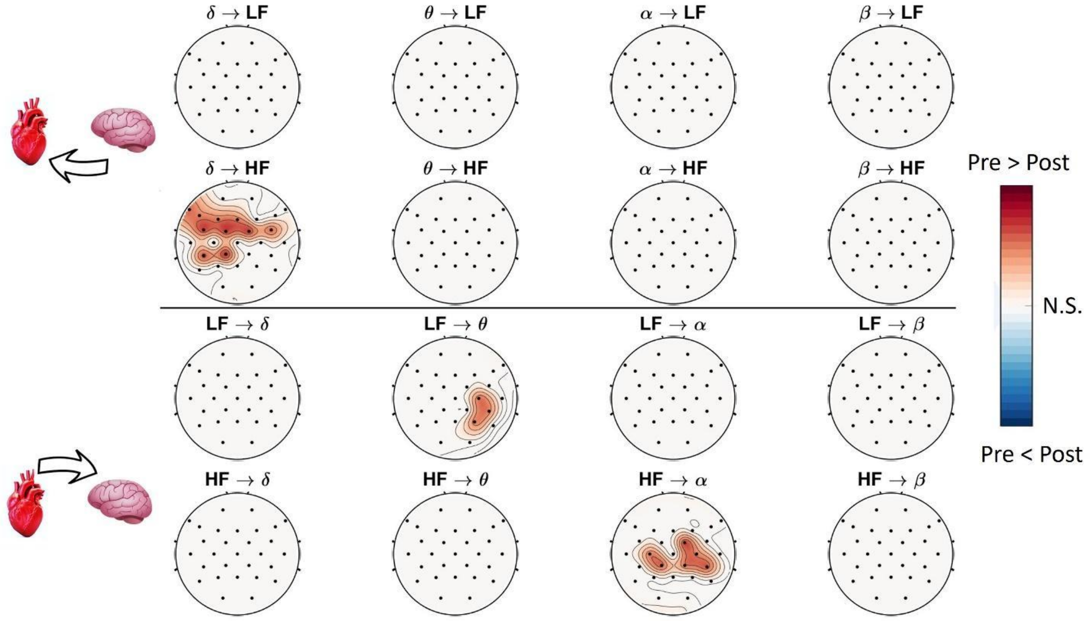

(d). Topographic maps illustrating the statistical results of the BHI analysis, comparing preictal and postictal periods using 1-minute windows.

Figure S1. Topographic maps illustrating the statistical results of the BHI analysis comparing preictal and postictal periods using different time windows than the original 10-minute one. The following windows are reported: (a) 7-minute, (b) 3-minute, (c) 2-minute, (d) 1-minute. For all figures, the first two rows are related to Brain-to-Heart interactions, for HRV-LF (first row) and HRV-HF (second row) bands, and for the examined brain waves (i.e., δ, θ, α, and β, represented in the four columns). The last two rows represent Heart-to-Brain interactions. White areas are not significant (N.S.), whereas red regions indicate significantly higher preictal values compared to postictal values, and blue areas indicate the opposite.

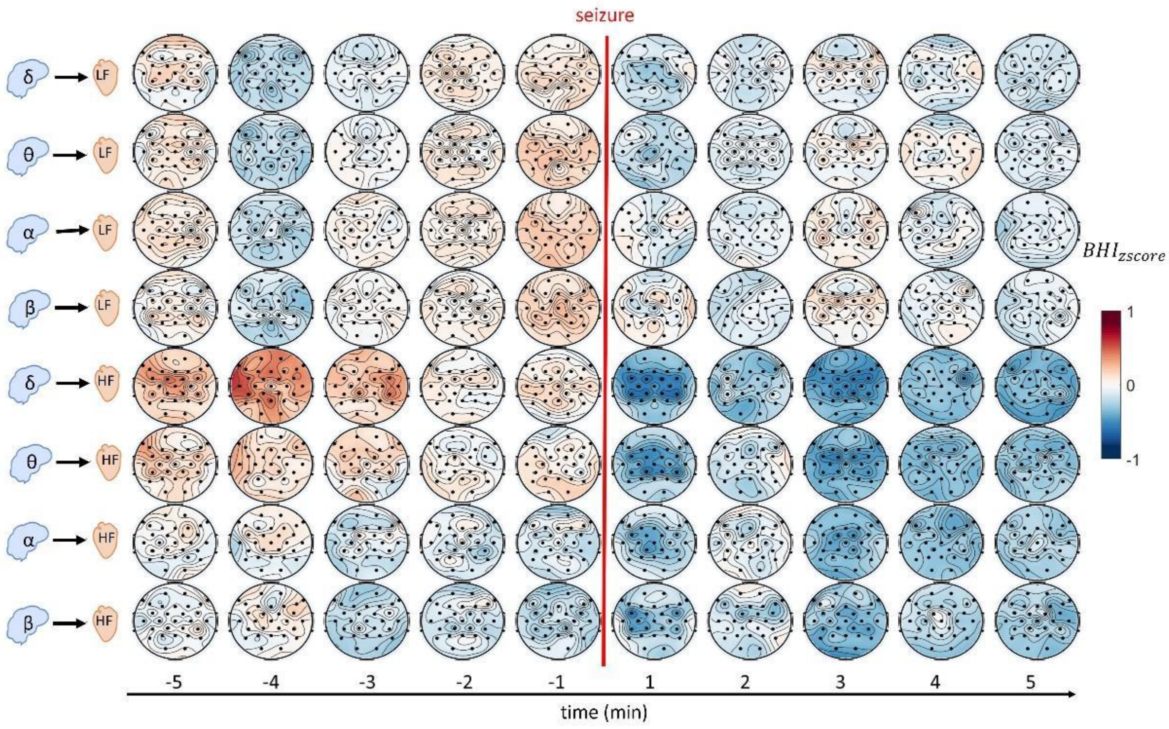

(a) BtH spatio-temporal dynamics.

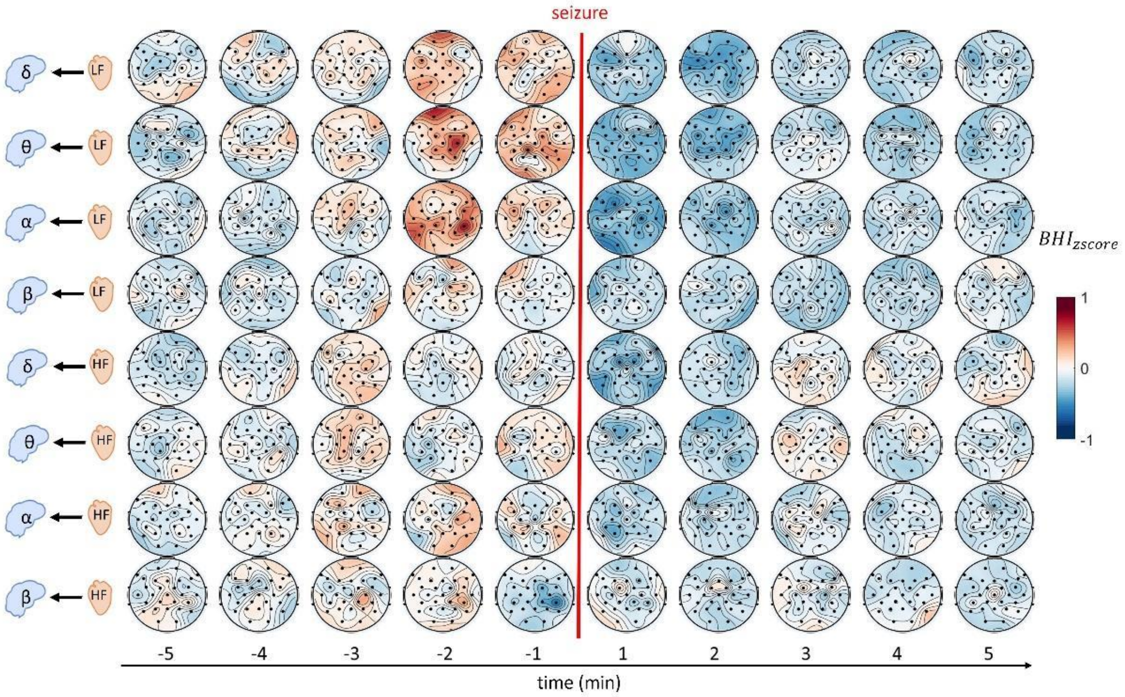

(b) HtB spatio-temporal dynamics

Figure S2. Each row represents the related BHI dynamics across the scalp reported in Figure 1 obtained as explained in Section Statistical analysis and topographic representation of BHI values. (a) BtH dynamics, (b) HtB dynamics.

Figure S3 displays the statistical outcomes of the BHI analysis conducted between two periods distant from the first ictal event for each recording. For this analysis, 5-minute windows were utilized, focusing on periods occurring 1 hour before the first ictal event. These 5-minute windows were intentionally separated by 1 minute, simulating a surrogate ictal event. This test was incorporated to assess whether random periods far from the ictal events might demonstrate differences in BHI analysis, similar to the statistical results presented in Figure 1 comparing preictal and postictal periods. In this instance, we considered a total of 28 events, taking into account the available interictal data in the Siena Dataset.

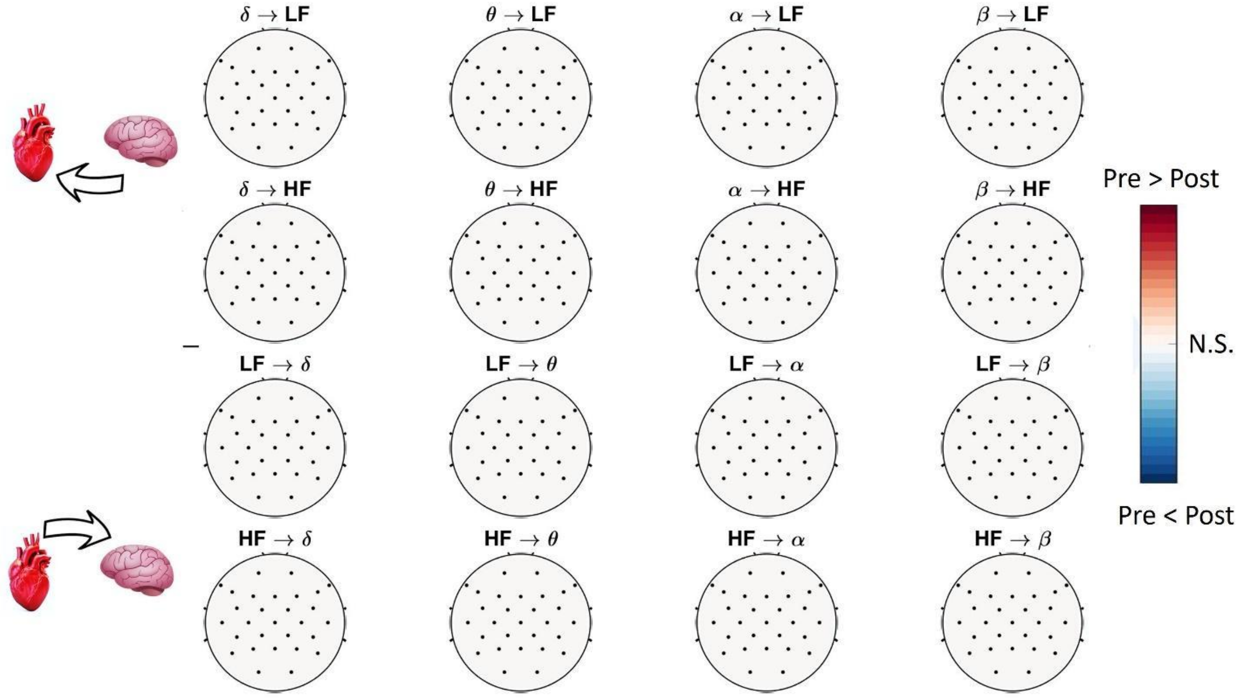

Figure S3. Topographic maps illustrating the statistical outcomes of BHI analysis comparing two 5-minute windows, segmented 1 hour before the first ictal event. The 5-minute windows are spaced 1 minute apart, simulating a surrogate ictal event, akin to the tests depicted in Figure 1. The first two rows pertain to Brain-to-Heart (BtH) interactions, focusing on HRV-LF (first row) and HRV-HF (second row) bands, and exploring brain waves (i.e., δ, θ, α, and β) represented in the four columns. The last two rows represent Heart-to-Brain (HtB) interactions. White areas indicate non-significant findings (N.S.).

In Figure S4, the statistical outcomes of BHI analysis comparing distant periods with preictal and postictal periods, as detailed in Figure 1, are presented. Each distant period corresponds to an interictal 10-minute window positioned 1 hour before the first ictal event. In this scenario, taking into account the available interictal and postictal data in the Siena Dataset, a total of 25 events were included in the analysis.

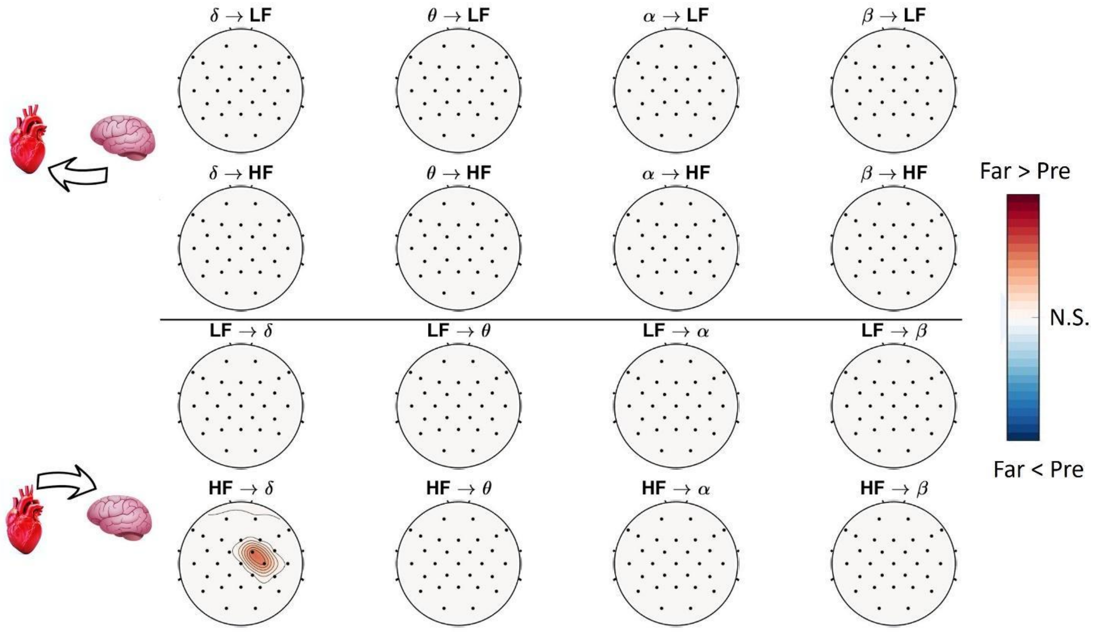

(a). Far interictal periods vs. preictal periods.

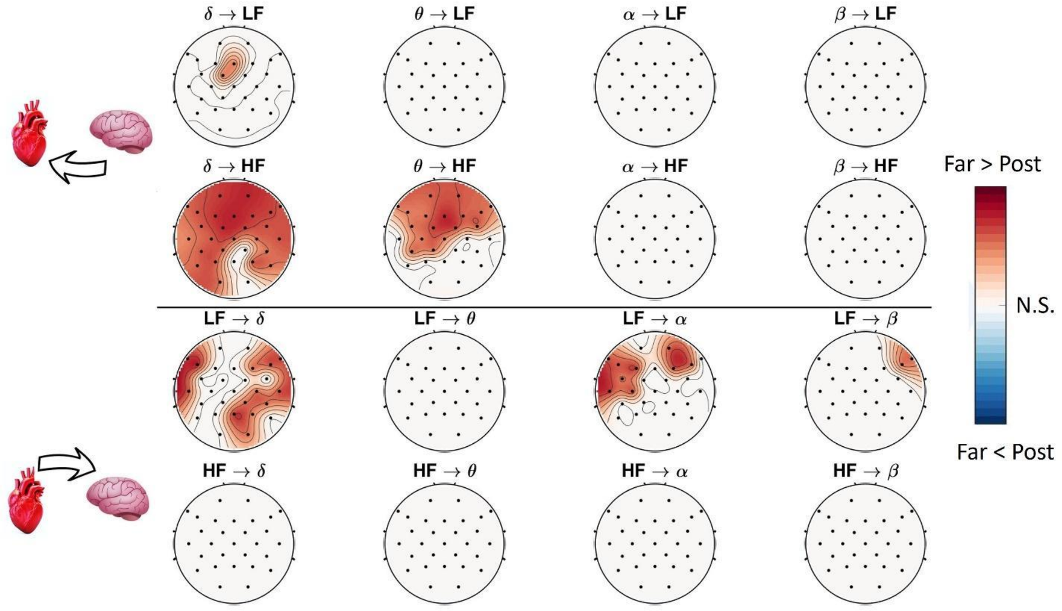

(b). Far interictal periods vs. postictal periods.

Figure S4. Topographic maps illustrating statistical results of BHI analysis comparing distant periods (i.e., a 10-minute window 1 hour before the first ictal events for each recording) versus (a) preictal periods and versus (b) postictal periods. For both figures, the first two rows pertain to Brain-to-Heart (BtH) interactions, exploring HRV-LF (first row) and HRV-HF (second row) bands, and examining brain waves (i.e., δ, θ, α, and β, represented in the four columns). The last two rows represent Heart-to-Brain (HtB) interactions. White areas indicate non-significant (N.S.) findings, while red regions indicate significantly higher distant values compared to (a) preictal values or (b) postictal ones, and blue areas indicate the opposite.
